# High-Throughput Microfluidic 3D Cytotoxicity Assay for Cancer Immunotherapy (CACI-IMPACT Platform)

**DOI:** 10.3389/fimmu.2019.01133

**Published:** 2019-05-28

**Authors:** Dohyun Park, Kyungmin Son, Yunchan Hwang, Jihoon Ko, Younggyun Lee, Junsang Doh, Noo Li Jeon

**Affiliations:** ^1^Division of WCU Multiscale Mechanical Design, Seoul National University, Seoul, South Korea; ^2^Department of Electrical Engineering and Computer Science, Seoul National University, Seoul, South Korea; ^3^Department of Materials Science and Engineering, Seoul National University, Seoul, South Korea; ^4^Department of Mechanical and Aerospace Engineering, Seoul National University, Seoul, South Korea; ^5^Institute of Bioengineering, Seoul National University, Seoul, South Korea; ^6^Institute of Advanced Machines and Design, Seoul, South Korea

**Keywords:** cytotoxicity assay, microfluidics, cancer immunotherapy, cytotoxic lymphocytes, high-throughput screening

## Abstract

Adoptive cell transfer against solid tumors faces challenges to overcome tumor microenvironment (TME), which plays as a physical barrier and provides immuno-suppressive conditions. Classical cytotoxicity assays are widely used to measure killing ability of the engineered cytotoxic lymphocytes as therapeutics, but the results cannot represent the performance in clinical application due to the absence of the TME. This paper describes a 3D cytotoxicity assay using an injection molded plastic array culture (CACI-IMPACT) device for 3D cytotoxicity assay to assess killing abilities of cytotoxic lymphocytes in 3D microenvironment through a spatiotemporal analysis of the lymphocytes and cancer cells embedded in 3D extra cellular matrix (ECM). Rail-based microfluidic design was integrated within a single 96-well and the wells were rectangularly arrayed in 2 × 6 to enhance the experimental throughput. The rail-based microstructures facilitate hydrogel patterning with simple pipetting so that hydrogel pre-solution aspirated with 10 μl pipette can be patterned in 10 wells within 30 s. To demonstrate 3D cytotoxicity assay, we patterned HeLa cells encapsulated by collagen gel and observed infiltration, migration and cytotoxic activity of NK-92 cells against HeLa cells in the collagen matrix. We found that 3D ECM significantly reduced migration of cytotoxic lymphocytes and access to cancer cells, resulting in lower cytotoxicity compared with 2D assays. In dense ECM, the physical barrier function of the 3D matrix was enhanced, but the cytotoxic lymphocytes effectively killed cancer cells once they contacted with cancer cells. The results implied ECM significantly influences migration and cytotoxicity of cytotoxic lymphocytes. Hence, the CACI-IMPACT platform, enabling high-throughput 3D co-culture of cytotoxic lymphocyte with cancer cells, has the potential to be used for pre-clinical evaluation of cytotoxic lymphocytes engineered for immunotherapy against solid tumors.

## Introduction

Adoptive transfer of *ex vivo* cultured/engineered cytotoxic lymphocytes (CLs) is arising as a promising approach to treat cancers (1). In particular, T cells expressing chimeric antigen receptor (or CAR-T cells) have been extremely successful in the treatment of CD19 expressing leukemia and lymphoma (2–4). The success has led to FDA approval of two CAR-T cell-based therapies, Kymriah (Novartis) and Yescarta (Gilead), and new CAR engineering strategies have been studied to improve the performance, reduce toxicity, and broaden applications of CAR-T therapy (5, 6). In addition, NK cells and γδ T cells, which exhibit low cytotoxicity and minimum graft-vs. -host disease in allogeneic transfer compared with T cells, have been developed as alternatives of CAR-T cells as an off-the-shelf therapeutics (7, 8). In spite of these efforts, the performance of adoptive transferred CLs against solid tumors is still limited due to complex tumor microenvironment (TME) that limit trafficking and effector functions of CLs (9, 10). In addition to highly immuno-suppressive microenvironments caused by acidic and hypoxic conditions and enrichment of suppressive cells (11–13), fibrotic tumor stroma is an important factor limiting successes of cancer immunotherapy by acting as a physical barrier for CLs to access tumor cells (14, 15). Therefore, various factors comprising TME need to be considered for the development of engineered CLs for solid tumors.

Cytotoxicity assay measuring killing ability of CLs is one of the most critical assays for the development of CLs for cancer immunotherapy. Chromium or calcein release assay based on the measurement of released radioactive ^51^Cr or fluorescence calcein from lysed cancer cells has been a standard method for assessing cell-mediated cytotoxicity (16, 17). These methods have been widely used because cytotoxicity can be assessed simply by co-culturing CLs with tumor cells loaded with ^51^Cr or calcein. In addition, these assays are compatible with 96 well formats, thus can be performed in high-throughput fashions. However, in these assays, tumor cells are either adhered on flat surfaces or suspended in medium, thus complex TME in solid tumors limiting CL activity are lacking.

Microfluidic-based platforms, which allow the reconstitution of complex 3D microenvironments of human tissues in *in vitro* by compartmentalization of multiple cell types, applying chemical and mechanical stimulations, and controlling chemical gradient (18), can be a powerful method for the assessment of lymphocyte cytotoxicity for solid tumors (19). Recently, microfluidic chips based on poly(dimethyl siloxane) (PDMS), a common material for microfluidics, were developed as preclinical models to evaluate antitumor activities of engineered T cells expressing T cell receptors specific for tumor antigens (or TCR-T cells) (20, 21) or engineered NK cells expressing Fc receptors (22) in 3D microenvironments recapitulating various aspects of TME. Specifically, microfluidic chips compartmentalized with 3D ECM gels containing tumor cells and TCR-T cell loading zones were used to assess the roles of hypoxia, inflammatory cytokines, immunosuppressive conditions induced by mTOR inhibitors, and monocytes on the cytotoxicity of TCR-T cells (20, 21), and microchips filled with ECM gels containing tumor cell spheroids and perfusable tubular vasculatures were used to recapitulate TME for NK cell trafficking toward solid tumors and to test combination of immuno-stimulatory biologics with NK cell therapy (22). However, the devices aforementioned requires labor and time intensive batch fabrication processes because the device was made of PDMS (23).

In this study, we introduce a 3D cytotoxicity assay using an injection molded plastic array culture (CACI-IMPACT) platform with which we can monitor both migration and cytotoxic activity of CLs in 3D microenvironment, by customizing our previous IMPACT device (24). We adopted extracellular matrix (ECM), which is a basic component of TME and did not exist in the standard protocols of cytotoxicity assays. ECM acted as physical barrier to restrict CLs from access cancer cells embedded in it. The limited accessibility resulted in low cytotoxicity compared with 2D assay. In addition, fibrotic ECM of TME was reconstituted by using denser collagen which lowered migration and cytotoxicity in observation of large area, but induced faster lysis process than sparser ECM. Furthermore, we improved the assay throughput compared with PDMS devices due to enhanced productivity oriented by changing material and usability mediated by rail-based microstructures. This model allowed us to test the effect of the physical properties of the 3D microenvironment on cytotoxic activity and we expect that this model can be used for high-throughput screening platform for estimating the efficacy of engineered lymphocytes in more *in vivo* like environment than conventional assays.

## Materials and Methods

### Cell Culture

HeLa cells were cultured in Dulbecco's modified eagle's medium (DMEM) with 10% of fetal bovine serum (FBS) and 1% of penicillin–streptomycin (PS). NK-92 cells were cultured in minimum essential media alpha (MEM α) with 15% of FBS, 15% of horse serum (HS), and 1% of PS and other supplements, including myo-inositol (0.2 mM), 2-mercaptoethanol (0.1 mM), folic acid (0.02 mM). NK-92 cells were sub-cultured in every 2 days in 6 ml of the full medium and 1,200 units of Interleukin-2. Sub-culture was conducted in T25 flasks with cell concentration of 10^5^ cells/ml.

### Fluorescent Labeling of Live and Dead Cells

HeLa cells were labeled with CellTrace™ CFSE Cell Proliferation Kit (Thermo fisher, C34570) by incubating the cells in serum free DMEM with 2 μM of CFSE for 30 min. NK-92 cells were labeled with CellTrace™ Far Red Cell Proliferation Kit (Thermo fisher, C34572) by incubating the cells in serum free MEM α with 2 μM of the reagent for 20 min. To detect dead cells, propidium iodide (PI) was used. For live imaging, PI-containing medium was used from the beginning of imaging. For imaging after 24 h of NK-92/HeLa co-culture, the medium was replaced with PI-containing medium for 30 min prior to imaging.

### 3D Cytotoxicity Assay Using Gel Patterned Device

CFSE-labeled HeLa cells were mixed with collagen gel pre-solution, which is a mixture of rat tail oriented collagen type I (Corning, 354249) with concentration of 9 mg/ml and 150 mM HEPES buffer at 2:1 ratio (v/v). The collagen pre-solution containing HeLa cells were patterned under the low rails of a CACI-IMPACT device (**Figure 2a**) following air plasma treatment with 70 W for 3 min. The device was incubated in a cell culture incubator with 37°C and 5% of CO_2_ for 30 min to crosslink the collagen. The cross-linked collagen gel blocks were immersed in media and further incubated for 24 h in the cell culture incubator. NK-92 cell suspension (2 × 10^6^ cells/ml, 2 μl) was loaded into the channel, and the device was stored in the incubator for 20 min at an angle of 90 degrees to let the NK-92 cells settle down on one side of the collagen block encapsulating HeLa cells. Then, the two medium reservoirs were filled with MEMα, and the devices were stored in the incubator for 24 h, or in a live imaging system. PI was added in the medium, and fluorescence images were acquired to assess cytotoxicity.

### Image Analysis

Time-lapse images were acquired using an inverted microscope system (Nikon eclipse Ti-E). Endpoint images of NK-92/HeLa co-culture were acquired using a confocal microscope (Nikon Ti 2 A1) through optical z-sectioning (depth: 100 μm, interval: 4 μm). For image analysis, we used Fiji. Z-projected images were used for display and the images were converted into binary images using auto threshold (“Mean” method) in Fiji for quantitative analysis. The number of NK cells in each sub-region was estimated by dividing the total area of NK cells into the average single NK cell area. Similarly, the percentage of killed HeLa cells were estimated using HeLa cell areas. Since live HeLa cells exhibited extended morphology whereas dead HeLa cells were rounded, areas of dead HeLa cells were converted to those of equivalent number of live HeLa cells by multiplying an average ratio of single live and dead HeLa cell area. Finally, the percentage of killed HeLa cells were calculated by (converted dead HeLa cell area)/(live HeLa cell area + converted dead HeLa cell area). Five unbiased students manually selected 20 NK and HeLa cells, respectively, and the average areas of the selected 100 NK and HeLa cells were used for single cell area of each cell type.

### 2D Cytotoxicity Assay

CFSE-labeled HeLa cells were plated in each well of a 24 well-plate (6 × 10^4^ cells/well) and cultured for 24 h. Then, various numbers of NK-92 cells were added to the well, and HeLa and NK-92 cells were co-cultured for another 24 h. PI was added to the media to have final concentration of 3 μM, and the percentage of dead HeLa cells were measured by fluorescence microscopy.

## Results

### Design and Fabrication of CACI-IMPACT Devices for Compartmented Hydrogel Patterning

**Figure 1:**
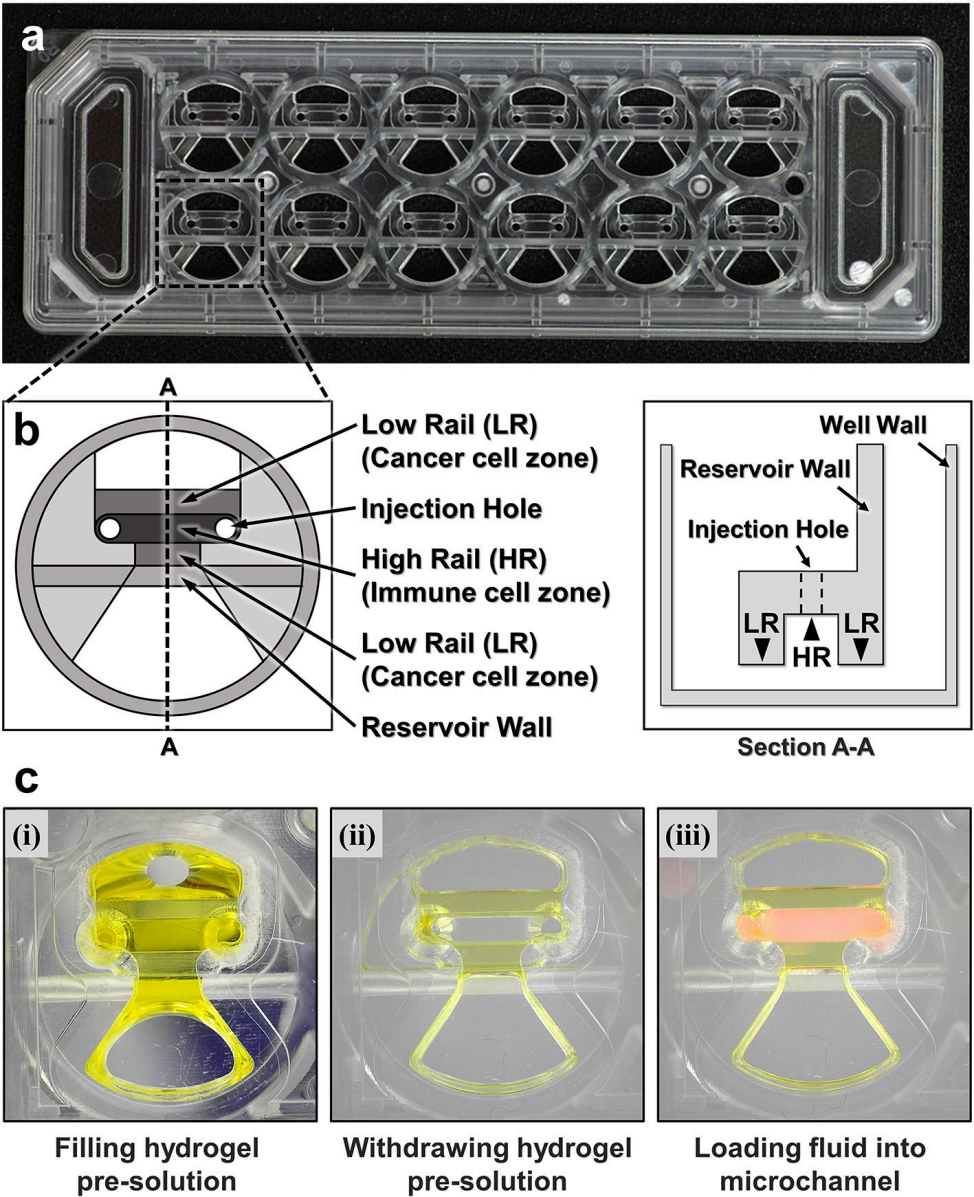
A CACI-IMPACT platform and its working process. **(a)** Rail-based microstructures are embedded in microwells with 96 well plate format and the structures are integrated in a 2 × 6 rectangular array. Water tanks are allocated in both sides to maintain humidity in samples. **(b)** Schematic top and section view of a single well. The microstructure in a single well consists of two low rails (LRs) for primary hydrogel patterning and one high rail (HR) to form a channel for secondary fluid patterning after hydrogel cross-linking. **(c)** Procedure of using the device. Once a hydrogel pre-solution is filled and withdrawn through an injection hole, the solution remains only underneath LRs. When the hydrogel is cross-linked, a microfluidic channel is formed where another fluid can be loaded.

To fabricate microfluidic devices for cytotoxicity assays in 3D ECM gels, we first designed and fabricated injection molded microfluidic devices that enable facile hydrogel patterning (Figure 1). To efficiently observe cytotoxic activity of CLs through fluorescence microscopes equipped in typical biology labs, the device was designed to have the same dimension as a standard microscope slide (3” × 1”), and rail-based microstructures under which cytotoxicity assays would be conducted were embedded in 2 × 6 rectangular array of wells with the same pitches as the conventional 96 well-plate (Figure 1a). The rail-based microstructures for hydrogel patterning is composed of two primary patterning rails (low rail, or LR), which are 100 μm apart from the bottom surface, and one secondary patterning rail (high rail, or HR), 500 μm apart from the bottom surface (Figure 1b). The rail-based microstructures allowed spatially compartmented hydrogel patterning to be performed by a simple and fast patterning process (Figure 1c). First, the surfaces of the device are hydrophilically modified via air plasma treatment. Next, hydrogel pre-solution was injected through an injection hole to fill the entire microstructures (Figure 1c-(i)), and subsequently aspirated away by pipetting. Due to the hydrophilicity of the surfaces, only hydrogel pre-solution underneath LR regions remained (Figure 1c,ii). Importantly, this process can be performed for 10 wells in a slide within 30 s (Movie 1). After crosslinking the hydrogel underneath LR regions, the second solution was loaded underneath the HR region to form two separate compartments (Figure 1c,iii). Compared with the PDMS devices widely used in microfluidics that requires tedious batch processes for fabrication, this injection molding-based device can substantially enhance throughput of the assay because the devices can be massively produced. In addition, hydrophilic rail-based microstructures permit hydrogel patterning to be conducted by simple pipetting, thus entire devices can be readily fabricated without requiring any sophisticated equipment/techniques.

### Cytotoxicity Assay in 3D ECM Environment

**Figure 2:**
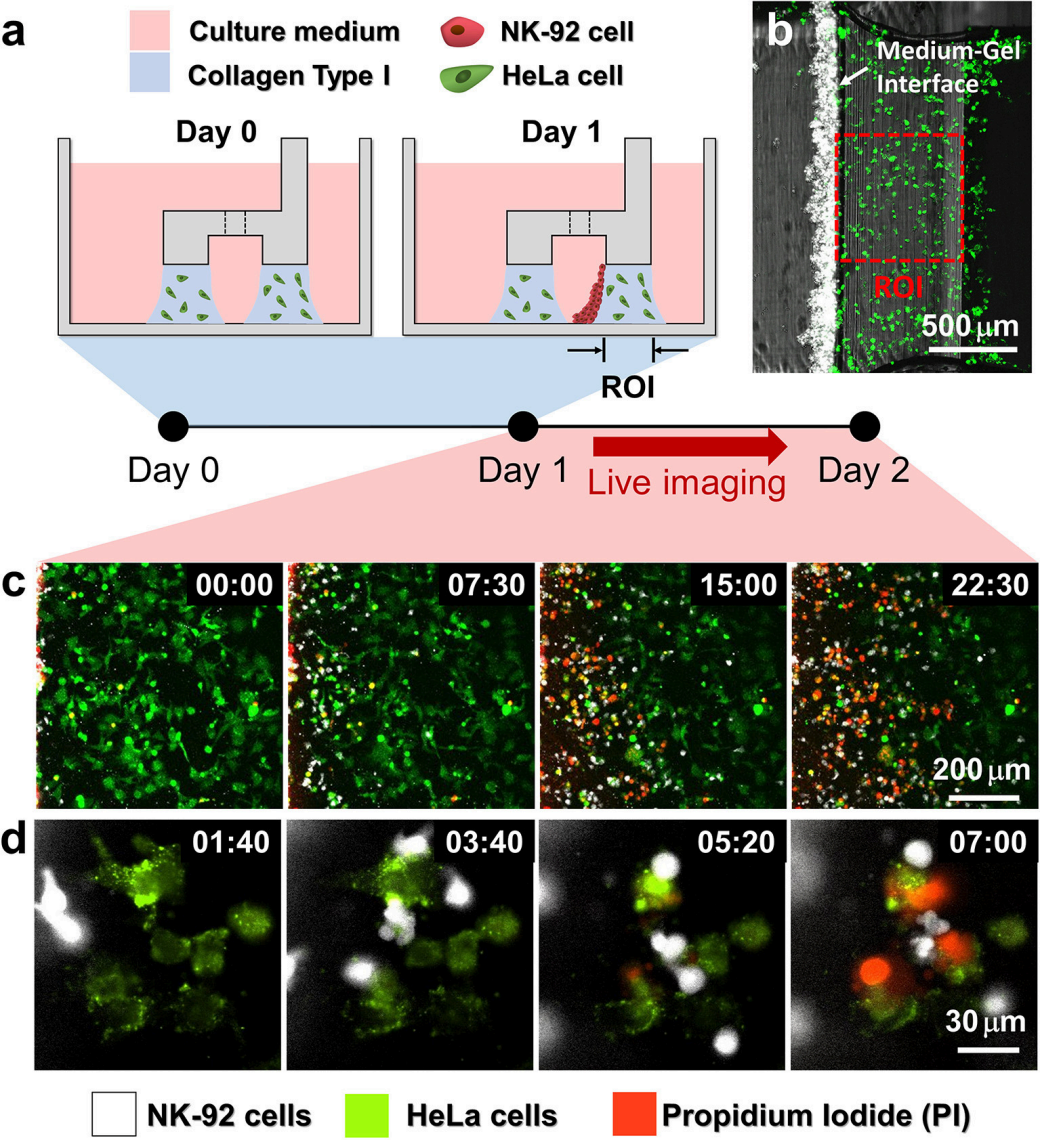
Procedure of 3D cytotoxicity assay and its outputs. **(a)** Schematic process of the assay. HeLa cells embedded in collagen were patterned under low rails (Day 0). After 24 h of cultivation, NK-92 cells were loaded into a microchannel formed by the hydrogel. By tilting the device at an angle of 90°, NK-92 cells were deposited on a collagen block (Day 1) and cultured for additional 24 h to observe migration and cytotoxic activity of NK cells. **(b)** Initial state of the assay (Day 1). **(c,d)** Live monitoring of migration and cytotoxic activity of NK-92 cells. Time is indicated in HH:MM in the top right corner of each image. See also Movies 2, 3.

Using the hydrogel patterning technique, we first fabricated collagen gels encapsulating HeLa cells underneath LR regions (Figure 2a Day 0**)**, and cultured for 1 day. Then, NK-92 cell suspension was loaded next to the collagen gel, and the device was tilted to 90° for 20 min to accumulate NK-92 cells on one side of the collagen gel by sedimentation (Figure 2a Day 1). NK-92 cells attached on the collagen gel surfaces penetrated into collagen gel blocks and migrated toward HeLa cells to exert cytotoxicity.

HeLa cells and NK-92 cells were labeled with two distinct fluorophores, and propidium iodide (PI), a fluorescence dye labeling dead cells, was added in the media to visualize dead cells. In this way, we can simultaneously observe NK-92 cell migration and cytotoxicity along the collagen matrix containing HeLa cells by live imaging (Figures 2b,c). NK-92 cells uniformly deposited on collagen block at the beginning of imaging penetrated and migrated into collagen matrix as shown in time-lapse images acquired with a low magnification objective lens (Figure 2b and Movie 2). As NK-92 cells propagated into collagen gels, PI-stained HeLa cells near NK-92 cells increased, meaning NK-92 cells exerted cytotoxicity against HeLa cells in collagen gels. Detailed procedures in CL-mediated cytotoxicity of cancer cells, rounding by detachment and subsequent membrane permeabilization allowing PI incorporation (25), can be visualized by time-lapse images using a high magnification objective lens: CFSE-labeled HeLa cells turned round after they made contact with NK-92 cells, followed by PI uptake (Figure 2c and Movie 3).

### 3D ECM Reduce Cytotoxicity by Limiting Cancer Cell Accessibility

**Figure 3:**
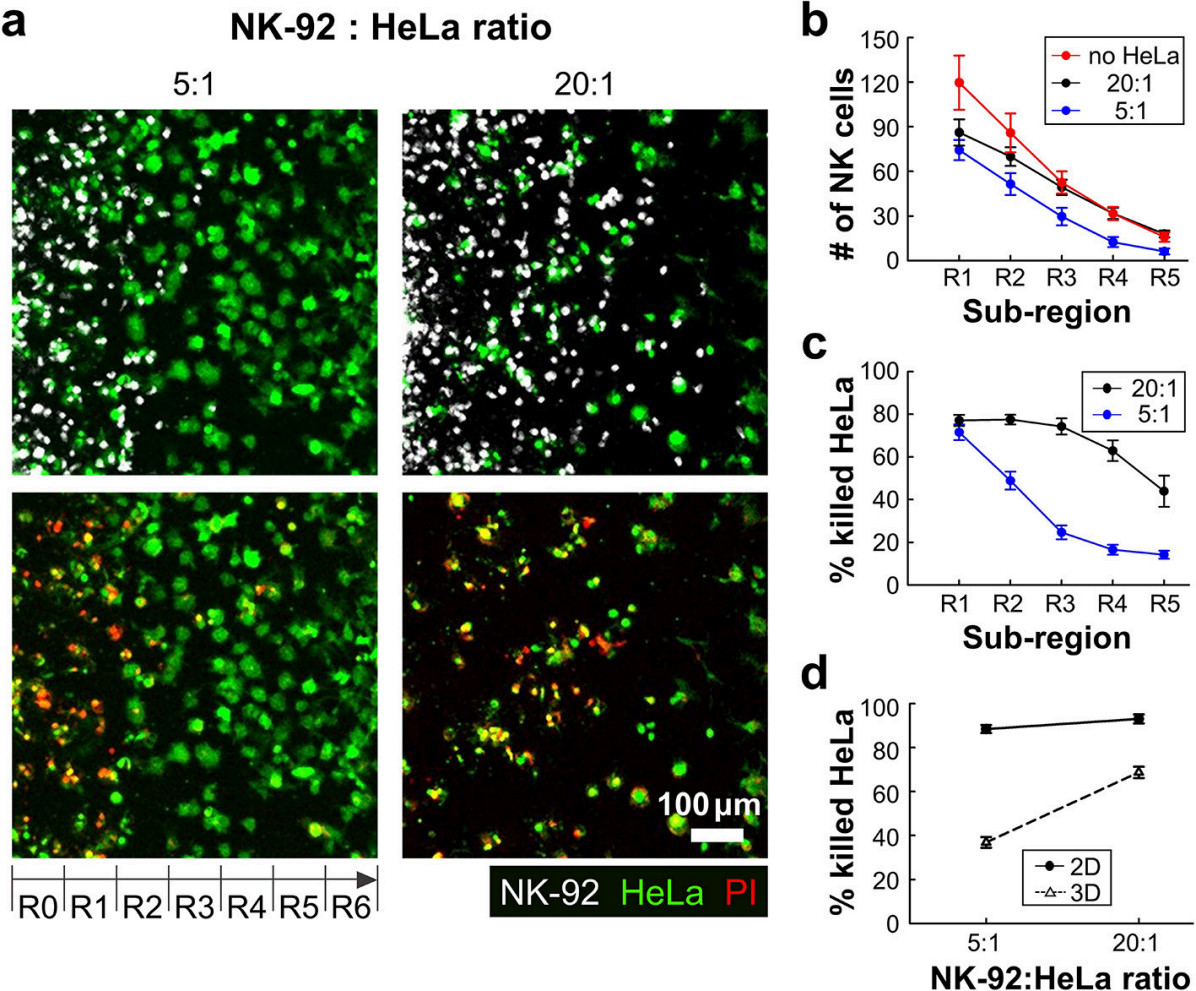
3D ECM reduces cytotoxicity by limiting cancer cell accessibility. **(a)** Images taken after 24 h of interaction of NK cells and HeLa cells in two NK-92:HeLa ratios. HeLa cells (green) and NK-92 cells (white) are displayed in upper images and lower images show live/dead HeLa cells at the same moment with the upper images. **(b)** The numbers of NK cells within the ROI sub-regions in collagens without HeLa cells (no HeLa), with 20:1 and 5:1 of NK-92:HeLa ratios (*n* ≥ 12). **(c)** The percentage of killed HeLa cells within the ROI sub-regions in the two NK-92:HeLa ratios (*n* ≥ 18). **(d)** The percentage of killed HeLa cells within the whole ROI from R1 to R5 in the two NK-92:HeLa ratios. (*n* = 3 for 2D assay, *n* ≥ 18 for 3D assay). Dot plots in **(b–d)** show mean ± SEM.

With this experimental setting, we first investigated how the presence of ECM and the density of cancer cells influenced NK cell cytotoxicity. HeLa cells in two different cell densities (0.8 × 10^6^ and 3.2 × 10^6^ cells/ml) were encapsulated in collagen gels (3 mg/ml) while the NK-92 cell density added in the media was fixed (2.0 × 10^6^ cells/ml), thus effectively total NK-92:HeLa were 5:1 and 20:1, typical ratio used for conventional cytotoxicity assays. Fluorescence images of square region of interest (ROI) with a side length of 700 μm, which is the dimension of the low rail width under which NK cells interact with HeLa cells, were acquired using a motorized stage 24 h after NK-92 cell seeding. The ROI was divided into seven sub-regions with a width of 100 μm, R0 to R6 (Figure 3a). The number of NK cells penetrated into the collagen gels and the percentage of killed HeLa cells, or PI-labeled HeLa cells, in each sub-region were measured and plotted (Figures 3b,c). R0 and R6 were not considered because they were located near the interface between collagen gel and liquid media where capillary force-mediated meniscus formed (Figure 3a), thus boundaries were not clearly defined in some cases.

NK-92 cell number was the highest in R1, and gradually decreased as the sub-region became deeper (Figure 3b). NK-92 cells exhibited significantly higher cell numbers in all sub-regions for the lower HeLa cell density, or 20:1, than the case of 5:1 except for R1 (Figure 3b). Similar trends were observed for HeLa cell killing, as HeLa cell killing requires close proximity of NK-92 cells and HeLa cells (Figure 3c). These results indicates that HeLa cells in collagen gels hold NK-92 cells nearby by forming dynamic immunological synapses (26), thus NK-92 cell migration toward deeper sub-regions gets delayed until they kill substantial fraction of HeLa cells. Percentages of killed HeLa cells in entire sub-regions R1-R5 were measured and compared with 2D cytotoxicity assays performed with equivalent NK-92:HeLa ratio. In 2D, NK-92 cells killed ~90% of HeLa cells regardless of the ratio, whereas significantly lower percentage of HeLa cells were killed in 3D, and the higher HeLa cell killing occurred with the NK-92:HeLa ratio of 20:1. These results indicate that accessibility of cancer cells is a limiting factor, and migration of CLs is a rate limiting step in cytotoxicity in 3D ECM microenvironments.

### Dense ECM Impede Migration of CLs but Facilitate Cancer Cell Lysis

**Figure 4:**
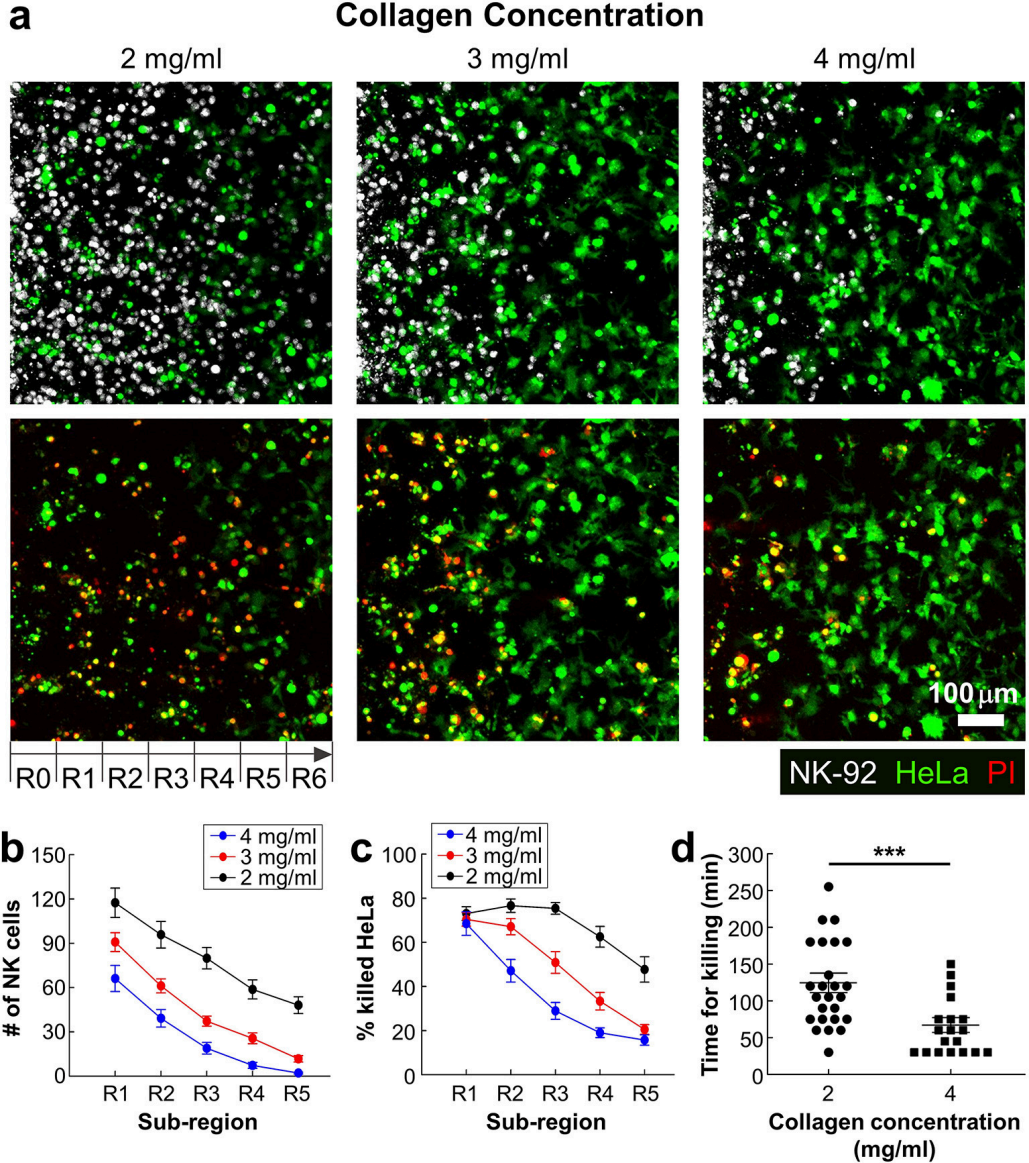
Dense ECM impedes cytotoxic activity of NK cells. **(a)** Images taken after 24 h of interaction of NK cells and HeLa cells in three collagen concentrations. HeLa cells (green) and NK-92 cells (white) are displayed in upper images and lower images show live/dead HeLa cells at the same moment with upper images. **(b)** The numbers of NK cells and **(c)** the percentage of killed HeLa cells within the ROI sub-regions (*n* ≥ 16). **(d)** PI uptake time from the moment that NK-92 cells contact with HeLa cells in 2 and 4 mg/ml of collagens. Each dot was obtained from a single HeLa cell killed by a single NK-92 cell. For statistical comparison, unpaired two-tailed Student's *t*-test was performed, and the statistical significance was ***p < 0.001.

In many solid tumors, fibrosis characterized by dense and stiff ECM generation occurs surrounding areas of tumor cells. Fibrosis not only affect cancer cells by triggering various mechanotransduction pathways by stiffening ECM (27), but also influence immunotherapy efficacy by limiting CL infiltration into tumors (9, 28). We sought to investigate the role of ECM density, a key component of fibrosis, on lymphocyte cytotoxicity in 3D by using the device patterned with various concentrations of collagen (2–4 mg/ml). Effective NK-92:HeLa ratio was fixed to 5:1. Representative still images of the patterned collagen gels with various collagen concentrations 24 h after NK-92 cell seeding are shown in Figure 4a. NK-92 cells distributed throughout the collagen gels in 2 mg/ml of collagen gel, whereas few NK-92 cells were observed in R4-R6 in 4 mg/ml of collagen gel. Overall, NK cell number was the highest in R1 and gradually decreased as the sub-region became deeper for all collagen concentrations (Figure 2b), and NK cell number was the highest for the lowest collagen concentration and gradually decreased as the collagen concentration increased for all sub-regions (Figure 2b). Similar trends in the percentage of killed HeLa cells were observed (Figure 2c). These results indicate that ECM density plays important role in NK cell migration in collagen gels, and consequently affect NK cell cytotoxicity.

Next, we investigated detailed interactions between NK-92 cells and HeLa cells encapsulated in different concentrations of collagen. By performing time-lapse imaging, we directly visualized NK-92 cell-HeLa cell interactions in a single cell level (Figure 2d and Movie 3 in SI), and assessed how much time is needed for each NK-92 cell for successfully killing HeLa cells. Time for killing, which measures time from initial NK-92/HeLa contact to PI uptake in HeLa cells, was measured for each NK-92 cell successfully killed NK-92 cell and plotted for NK-92 cells in collagen gels with 2 and 4 mg/ml (Figure 4d). Interestingly, time for killing of NK-92 cells in 2 mg/ml collagen gels was significantly lower than that of NK-92 cells in 2 mg/ml collagen gels. This result indicate that cytotoxicity of individual NK-92 cells is higher in higher concentration of collagen gels. Taken together, ECM density can influence both migration and cytotoxicity of CLs.

## Discussion

Traditional *in vitro* 2D cytotoxicity assays against cancer cells have been widely used to evaluate *ex vivo* engineered or cultured CLs due to simplicity in assays, but the assay results may not be consistent with *in vivo* results due to the absence of 3D tumor microenvironment (TME). PDMS-based microfluidic devices recapitulating various aspects of TME, including hypoxia, inflammatory cytokines, immunosuppressive conditions, and vasculatures, have been developed to evaluate CLs in 3D (20–22).

However, PDMS-based devices require labor and time intensive batch fabrication processes, thus device fabrication limits experimental throughputs (23). To overcome this limitation, the CACI-IMPACT devices used in this study were massively produced using injection molding with polystyrene (PS) by customizing the design of the IMPACT device (24), which was previously developed by our group for 3D compartmentalized cell culture. In our experience of conducting the same 3D cytotoxicity assays using our PDMS-based co-culture device (29), approximately 2 days of serial processes, including casting (7 h), punching (1 h), bonding (10 m), and surface hydrophobicity restoration (>1 d), were required. The series of manual processes can cause defects, resulting in lower yields or lower uniformity of the final devices to be used in experiments. Furthermore, pressure sensitive loading process in PDMS device reduces usability and experimental throughput. In sharp contrast, CACI-IMPACT device requires < 10 min for device preparation including 3 min of hydrophilic surface modification. In case that the device was packed after plasma treatment, no preparation is required except opening the packaging. Rail-based microstructures with hydrophilic surfaces further facilitated experiments by enabling simple and fast hydrogel patterning to be performed, and multi-well format further enhanced experimental throughputs by allowing multiple experiments to be performed simultaneously in a single device. In addition to improved device fabrication and experimental throughputs, long term monitoring of CL-cancer cell interactions is possible, as media change can be readily performed by aspirating media in the media reservoir and filling new media without perturbing hydrogels containing cells.

Using the injection molded devices, we performed 3D cytotoxicity assays with various density of cancer cells and various collagen concentrations. First, we found the presence of ECM and cancer cells could significantly reduce cytotoxicity of CLs by impeding migration and limiting accessibility of cancer cells compared with 2D cytotoxicity assays (Figure 3). Presence of cancer cells in ECM may play dual role in cytotoxicity: it may impede infiltration of CL by interacting with CLs as we have shown, but at the same time, cancer cells can promote CL migration by producing chemokines such as CXCL9, 10, and 11 (30). As shown in Figure 3b, NK-92 cell distribution in collagen gels lacking HeLa cells (no HeLa) was comparable to that in collagen gels with low density of HeLa cells (20:1) except for entry regions, where NK-92 cell numbers were slightly higher for collagen gels lacking HeLa cells, indicating chemotaxis-mediated NK-92 cell migration was minimal in our system. Second, we found ECM density played an important role in 3D cytotoxicity by independently regulating migration-mediated cancer cell contact and direct cancer cell killing (Figure 4). Indeed, collagen density can influence various physical properties of collagen gels such as pore size and stiffness (31). When collagen concentration was increased, NK cell infiltration into collagen gels was substantially reduced presumably due to reduced pore size that limits amoeboid-mode immune cell migration (32). Indeed, increased ECM density observed in fibrotic tumors reduced activity of T cells by limiting physical access of tumor cells (14), indicating our device may be a good model system to evaluate *ex vivo* engineered CLs for fibrotic tumors. Interestingly, cytotoxicity of individual NK cells were significantly enhanced when collagen concentration was increased (Figure 4d). While detailed mechanisms for enhanced cytotoxicity in collagen-dense environments need to be determined, it is possible that stiff ECM environments in high concentration of collagen facilitate tumor cell lysis by increasing tumor cell tension, which enhances perforin-mediated pore formation on tumor cell membrane (33).

Our preliminary 3D cytotoxicity assay using human primary NK cells revealed that CACI-IMPACT platform can be used for primary lymphocytes, while detailed assay conditions need to be adjusted depending on cell types (Figure S1). Primary NK cells exhibited much higher motility and cytotoxicity compared with NK-92 cells: they uniformly distributed in entire collagen gels (Figure S1b) and killed the majority of HeLa cells (Figure S1c) within 12 h in dense ECM (4 mg/ml of collagen), in which NK-92 cells killed only ~40% of HeLa cells for 24 h (Figure 4c). Superior cytotoxicity of primary NK cells in our assay is partly due to smaller size of primary NK cells (diameter ~8 μm) compared with that of NK-92 cells (diameter ~ 14 μm), which plays important role in cell migration in dense ECM (31), further confirming importance of lymphocyte motility in 3D cytotoxicity.

To sum up, we introduced an injection molded microfluidic device for assessing cytotoxicity of CLs in 3D environment. The proposed device is characterized by (i) enhanced productivity via injection molding, (ii) enhanced experimental throughput mediated by multi-well format of the device, and (iii) hydrophilic rail-based microstructures facilitating hydrogel patterning with simple pipetting. Using the device, we found 3D ECM significantly reduce cytotoxicity of CLs by impeding migration and access to tumor cells compared with traditional 2D assays. We also found denser ECM impede migration of CLs but enable effective killing once CLs contact with tumor cells. The results show how important the presence of ECM is for accessing cytotoxicity of CLs against solid tumors. We think this injection molded 3D culture platform could be used to evaluate cytotoxicity of CLs in 3D environment and to identify new therapeutic approaches mediated by adoptive transferred CLs against solid tumors.

## Data Availability

The raw data supporting the conclusions of this manuscript will be made available by the authors, without undue reservation, to any qualified researcher.

## Author Contributions

DP designed the study, conducted experimental work, analyzed the data, and wrote the paper. KS conceptualized the work and conducted experimental work. YH conducted experimental work and analyzed the data. JK and YL designed the device used in the study. JD and NJ supervised experimental work, data analysis, and wrote the paper.

### Conflict of Interest Statement

NJ is a founder of Curiochips inc., and he holds equity in this company. The CACI-IMPACT device is posted on the company website (https://www.curiochips.com/). The remaining authors declare that the research was conducted in the absence of any commercial or financial relationships that could be construed as a potential conflict of interest.

## Supplementary Material

Movie S1 Demonstration of high-throughput fluid patterning with green food dye mixed water.

Movie S2 Live monitoring of cytotoxic activity of NK-92 cells against HeLa cells within CACI-IMPACT platform acquired with a low magnification objective lens.

Movie S3 Live monitoring of single cell level cytotoxic activity of NK-92 cells against HeLa cells.

Figure S1 3D cytotoxicity assay performed with primary NK cells against HeLa cells. **(A)** Representative images taken after 6H (left) and 12H (right) of primary NK/HeLa cells co-culture. **(B)** The number of primary NK cells and **(C)** the percentage of killed HeLa cells within the ROI sub-regions after 6H (black) and 12H (red) of primary NK/HeLa cells co-culture.
