# Supplementary figures and images for: High-Throughput Microfluidic 3D Cytotoxicity Assay for Cancer Immunotherapy (CACI-IMPACT Platform)
Source: Front Immunol. 2019 May 28;10:1133. doi: 10.3389/fimmu.2019.01133 (PMC6546835; doi:10.3389/fimmu.2019.01133)

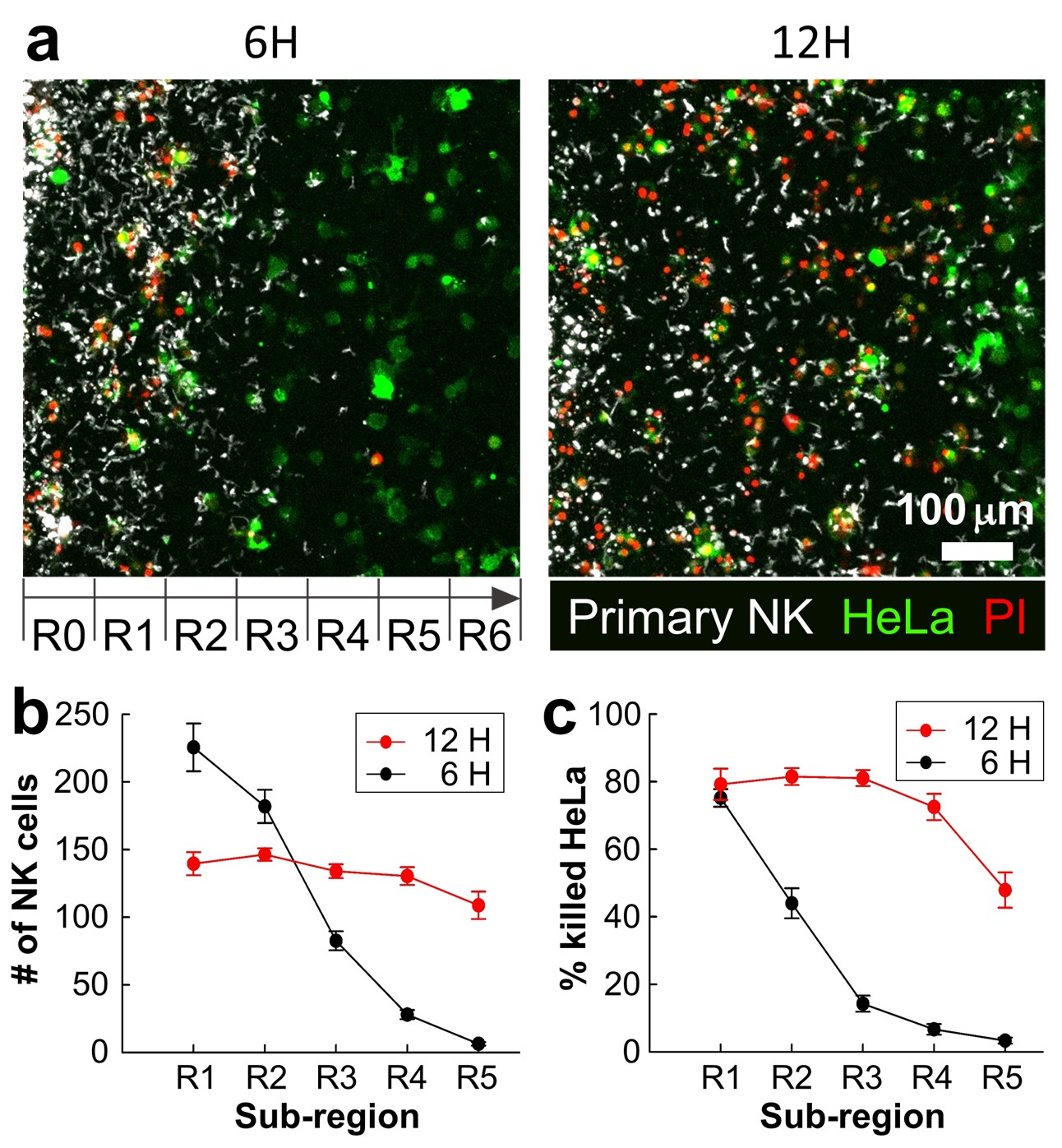

Supplement: Figure S1 — 3D cytotoxicity assay performed with primary NK cells against HeLa cells. (A) Representative images taken after 6H (left) and 12H (right) of primary NK/HeLa cells co-culture. (B) The number of primary NK cells and (C) the percentage of killed HeLa cells within the ROI sub-regions after 6H (black) and 12H (red) of primary NK/HeLa cells co-culture. [file Image_1.JPEG]
